# Gut microbiome and CAR-T therapy

**DOI:** 10.1186/s40164-019-0155-8

**Published:** 2019-11-19

**Authors:** Muhammad Bilal Abid, Nirav N. Shah, Theresa C. Maatman, Parameswaran N. Hari

**Affiliations:** 1 Division of Infectious Diseases, Medical College of Wisconsin (MCW), Hub for Collaborative Medicine, 8701 Watertown Plank Road, Milwaukee, WI 53226 USA; 2 Division of Hematology/Oncology, Medical College of Wisconsin (MCW), Milwaukee, WI USA; 3 Division of Internal Medicine, Medical College of Wisconsin (MCW), Milwaukee, WI USA

**Keywords:** Immunotherapy, Immuno-oncology, CAR T-cells, TRUCKs, Gut microbiome, Dysbiosis, CRISPR/cas9

## Abstract

Considerable progress has been made in cancer therapeutics recently with targeted strategies that are efficacious and less toxic. Immunotherapy and chimeric antigen receptor (CAR) T-cells are increasingly being evaluated in a variety of tumors in the relapsed/refractory as well as frontline disease settings, predominantly in hematologic malignancies (HM). Despite impressive outcomes in select patients, there remains significant heterogeneity in clinical response to CAR T-cells. The gut microbiome has emerged as one of the key host factors that could potentially be modulated to enhance responses to immunotherapy. Several recent human studies receiving immunotherapy showed a significantly superior response and survival in patients with the more diverse gut microbiome. Currently, it is unknown if gut microbiota modulates anti-tumor responses to CAR T-cells. Based on molecular and immunological understanding, we hypothesize that strategically manipulating gut microbiota may enhance responses to CAR T-cells. In this review, we further discuss resistance mechanisms to CAR T-cells in HM, potential approaches to overcome resistance by harnessing gut microbiota and other related novel strategies.

## Introduction

Tremendous advances have been made in cancer therapeutics recently with the advent of targeted immunotherapeutic approaches that are efficacious with potentially less toxicity than the standard-of-care chemotherapy. Both immunotherapy, via programmed cell death 1 (PD-1) receptor, programmed cell death ligand 1 (PD-L1) or cytotoxic T-lymphocyte antigen-4 (CTLA-4) inhibitors, and chimeric antigen receptor (CAR) T-cells have impacted the natural history of both solid and hematological malignancies (HM). These are increasingly being studied in a variety of tumors in patients with relapsed/refractory (R/R) malignancy [1–5]. CAR T-cells, currently approved in HM, are associated with detrimental on-target, off-tumor adverse effects such as cytokine release syndrome (CRS), neurotoxicity, and suppression of humoral immunity due to B-cell aplasia [2, 6]. However, the response to such individualized therapies remains heterogeneous [6–9]. One strategy to circumvent this is to better understand and, thereafter, modulate hosts’ factors. And gut microbiome is an environmental/host factor that has generated unprecedented excitement recently.

The role of the gut microbiota in health and disease, its bidirectional negotiation with the systemic immunity and the existing evidence of the critical role of the gut microbiome in immune checkpoint inhibition (ICI) responses and survival are summarized in Abid MB, 2019 [10].

## Potential of the gut microbiota in improving ICI responses

The results of pre-clinical and clinical studies thus far demonstrate an impact of the gut microbiota on ICI responses, both positive and negative. Predominantly observational, the existing evidence shows that diversity and compositional differences of the gut microbiome may influence response to ICI treatment (PD-1, PD-L1, and CTLA-4) and outcome [10, 11]. These results are encouraging and pave the way towards further prospective studies to unravel response modulation strategies to immune and cellular therapies and, possibly, potentiate their effect. Furthermore, anti-CTLA-4 antibodies enhance the anti-tumor effect by depletion of regulatory T-cells (Tregs) [12–14]. Hence, Tregs abundance in the tumor milieu may now be further curtailed by regulating the gut flora [10]. Immune-mediated adverse events may also be ameliorated with modifying gut flora. For instance, a few studies have shown that the adverse events due to checkpoint inhibition with ipilimumab were mediated by elevated levels of IL-17 [15, 16]. With a greater understanding of the gut microbiome and its impact on systemic immunity, we may be able to prevent such off-target effects by regulating Th17 differentiation via more regulated manipulation of gut microbiota.

## The potential for improved responses to CAR T-cells

CAR T-cells are autologous T-cells re-directed towards a tumor-specific antigen and are a successful modality for patients with refractory B-cell hematological malignancies. The intracytoplasmic and transmembrane portions of a CAR T-cell contain signaling domains that are involved in T-cell activation and durability, such as CD3ζ, CD28, 4-1BB, ICOS, and OX40. A characteristic second-generation CAR connects an antigen-binding domain, typically single-chain variable fragments (scFv) derived from a monoclonal antibody, to the T-cell signaling domain (CD3ζ: CD3 zeta-chain) coupled to the intracellular signaling domain of a costimulatory molecule such as CD28 or 4-1BB. Higher-generation CAR T-cell constructs may contain multiple signaling domains. These components, including the engineered receptor protein and signaling domains, then facilitate activation of CAR T-cells and render durability, thus allowing execution of the effector functions of a CAR T-cell independent of MHC restrictions [6, 17, 18]. The FDA approved CAR T-cells directed against CD19 [CD19+ CAR T-cells; tisagenlecleucel (Kymriah™, Novartis)] in August 2017 to treat acute lymphoblastic leukemia (ALL) and then axicabtagene ciloleucel (Yescarta™, Kite Pharma) for the treatment of R/R large B-cell lymphoma in October 2017 [19, 20]. Although these treatments prolong the survival of patients with relapsed and refractory diseases, the success rates remain heterogeneous and long-term durability of response has yet to be determined [6, 9, 17, 19, 20].

While there is no evidence on the impact of gut microbiome manipulation on CAR T-cell responses as of this writing, we hypothesize that gut microbiota modulation carries potential for enhancing CAR T-cell responses: based on pre-clinical and growing clinical evidence elucidating escape mechanisms in CAR T-cell non-responders, clinical evidence of enhancement of ICI responses with gut microbiota manipulation, and shared immunological features between CAR T-cells and ICI. We discuss below factors that impact CAR T-cell responses and known resistance mechanisms to CAR T-cell therapy. We further highlight potential immunologic associations based on which we postulate that the gut microbiota manipulation may be harnessed to homogenize CAR T-cell response.

### Pre-CAR conditioning and the role of antibiotics

Most adoptive T-cell transfer (ACT) modalities involve pre-conditioning with some form of lymphodepletion, either chemotherapy or irradiation. These pre-conditioning strategies induce microbial translocation that amplifies the function of effector T-cells [21–23]. A similar mechanism may potentially enhance adoptively transferred tumor-specific CD8+ T-cells via dendritic cell activation. Paulos et al. [22] showed that the immune-enhancing effects of irradiation were dampened in mice receiving antibiotics in a melanoma mice model. Xu et al. had earlier demonstrated similar results with cyclophosphamide and Viaud et al. further showed that antibiotic treatment dampened cyclophosphamide-mediated antitumor effects in a sarcoma mice model [21, 23]. In a separate murine study, Uribe-Herranz et al. [24] demonstrated that vancomycin-mediated depletion of *Bacteroides* spp. enhanced ACT function in an IL-12-dependent manner. This finding was further reiterated by a French study by Routy et al. involving 249 patients with advanced NSCLC, RCC and urothelial carcinoma receiving ICIs with anti-PD1 and anti-PDL1. They showed that patients who received antibiotics in the peri-ICI blockage period had shorter survival and metagenomic analysis revealed that *Akkermansia muciniphila* was enriched in responders. This effect was mediated by an increment in IL-12 and a concomitant decrease in Tregs in the TME [25, 26].

However, Vétizou et al. showed that other Bacteroides species, specifically *B. fragilis* and *B. thetaiotaomicron*, promoted the efficacy of CTLA-4 blockade in mice, in contrast to the *Bacteroides*-induced suppression of ACTs demonstrated by Uribe-Herranz et al. [24, 27]. Kuczma et al. [28] further showed that antibiotics diminished cyclophosphamide-induced endogenous T-cell responses in the ACT setting in a murine study. Interestingly, long-term antibiotics had no impact on the efficacy of CD19+ CAR T-cell although it impacted their long-term persistence. This paradoxical pre-clinical finding has not been reproduced as no other similar studies have been performed in the CAR T-cell setting yet.

As infections account for much of morbidity and mortality of patients undergoing chemo-immunotherapy, transplantation, and ACT, the use of prophylactic antibiotics is not uncommon. Although there are conflicting preliminary, pre-clinical data on the impact of antibiotic usage in the setting of ACT, substantial evidence exists on the positive impact of conditioning-induced endogenous T-cell responses. The preconditioning’s positive impact, taken together with broad-spectrum antibiotics-induced dysbiosis in the setting of chemo-immunotherapy, intermittent use of narrow-spectrum antibiotics may carry potential in strategically averting unfavorable taxa to maximize the efficacy of CAR T-cells. Further, large-scale studies will be needed in this setting before any change in practice.

### The fine balance between effector T-cells and immunosuppressive Tregs

Based on pre-clinical and human studies suggesting an association between antigen presentation machinery and gut microbial diversity, coupled with evidence suggesting a suppressive role of Tregs on ACTs, we postulate that modulating effector T-cell and Treg balance via gut microbiota may very well impact responses to CAR T-cells [29, 30].

**Fig. 1:**
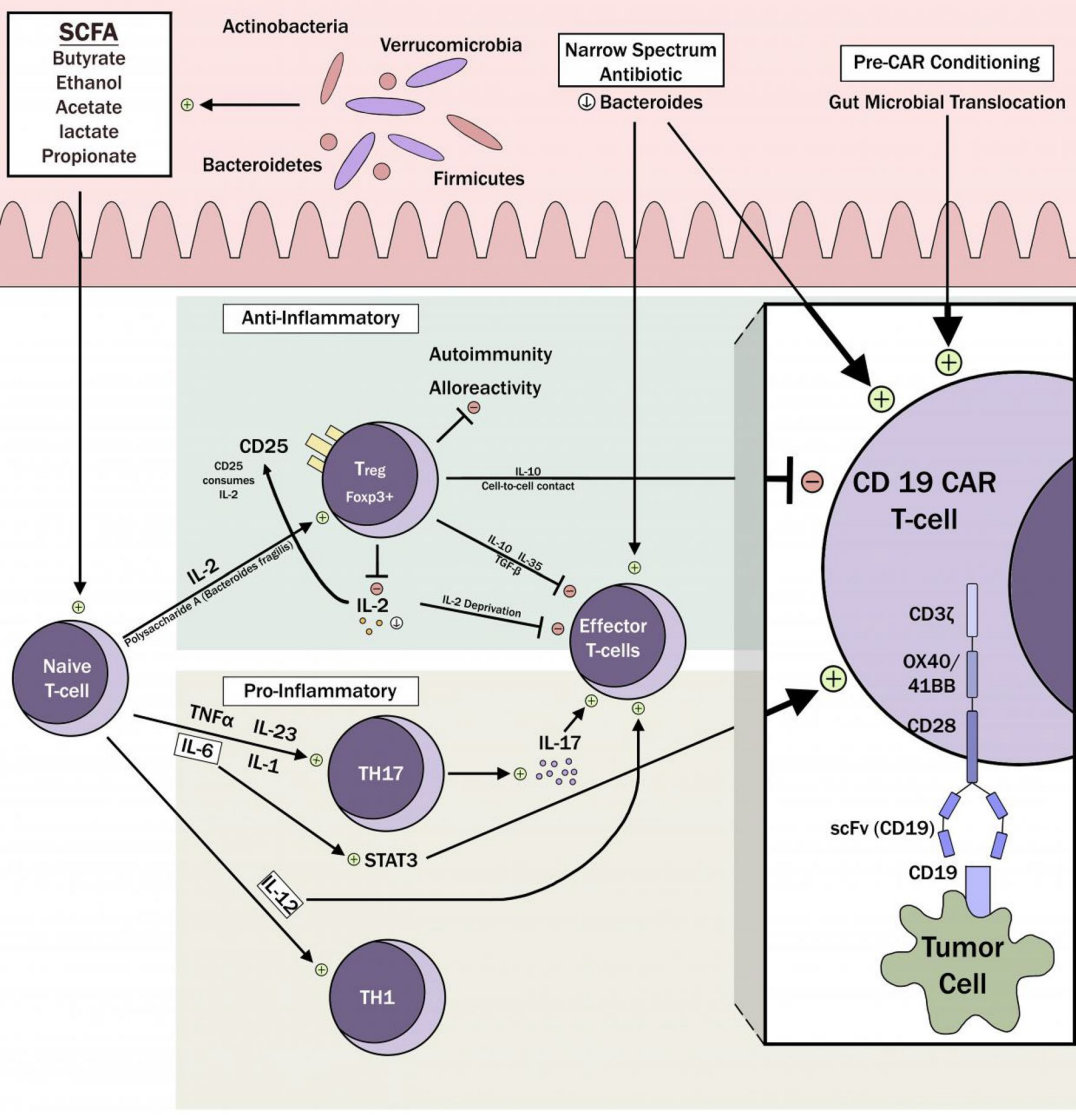
Gut microbiota mediates the differentiation of naïve T-cells either into pro-inflammatory Th17 or anti-inflammatory, Tregs. These effector T-cells then migrate to systemic circulation from mLN. Th17 boosts effector T-cells, mainly mediated via IL-17, whereas Tregs suppress effector T-cell function, mediated via IL-10. Specific gut taxa may potentially be harnessed to enhance CAR T-cell responses in several ways (figure’s left to right): By influencing pre-CAR conditioning; by using specific, narrow-spectrum antibiotics to deplete select, detrimental gut microbes; suppression of Foxp3+ Tregs and hence circumventing Treg-induced CAR T-cell suppression; upregulation of IL-6/STAT3 signature; direct activation of CAR T-cells (similar mechanism as that of endogenous T-cells)

Fraietta et al. proposed a model to predict response to CD19+ CAR T-cells in heavily pre-treated, high-risk chronic lymphocytic leukemia (CLL) patients based on baseline T-cell qualities. They showed an association between the upregulation of the IL-6/STAT3 signature and durable clinical remissions in CLL patients treated with CD19+ CAR T-cells [31] (hypothetical model: Fig. 1). This critical finding demonstrated a promising strategy to improve CAR T-cell responses by sorting/selection of lymphocytes before CAR transduction. It remains unknown if the gut microbiota could have any impact on the intrinsic transcriptome profile of a CAR-T cell but IL-6/STAT3 signature could be regulated via defined gut microbiota, for instance, at the time of CAR T-cell infusion [32].

With regards to the immunosuppressive effect of Tregs in ACT setting, a recent study by Duell et al. [30] suggesting a critical role of Tregs in dictating the response to bi-specific T-cell engagers (BiTEs) further pave the way towards a greater understanding of their role in ACTs. The exact mechanism remains to be explored but hypothesized mechanisms include either direct cell-to-cell contact, through inhibitory cytokines such as IL-10, IL-35, and TGF-B or ‘metabolically’ via high expression of CD25 which drives Tregs to consume local IL-2 and therefore starve CAR T-cells by depleting the IL-2 they need to survive [33] (Fig. 1). Cytokine-mediated adverse effects associated with CAR T-cells, such as CRS, neurological toxicity, and hypersensitivity, may potentially be mitigated in the future by manipulating gut microbiome such that it is skewed away from orchestrating proliferation of those Th subsets which are responsible for the excessive production of pathogenic cytokines.

### Inhibitory ligand upregulation and CAR T-cell exhaustion

Tumor-associated antigen (TAA) loss leading to CD19 escape is the commonest cause of resistance to CAR T-cells in ALL patients [20, 34, 35]. However, this only accounts for 10–20% of non-responders in lymphoma patients and even less so in other hematological malignancies such as CLL as well as solid tumors. The immunosuppressive TME created through the upregulation of inhibitory ligands by the tumor accounts for major hindrances in lymphoma and CLL patients [34, 36]. Interestingly, CAR T-cells demonstrate similar susceptibility to these inhibitory immune checkpoints as endogenous T-cells [36, 37]. Also, an increased PD-1 expression has been demonstrated in CAR T-cells in pre-clinical and clinical studies and is associated with increased T-cell exhaustion, another mechanism linked to CAR T-cells failure [36, 38].

**Fig. 2:**
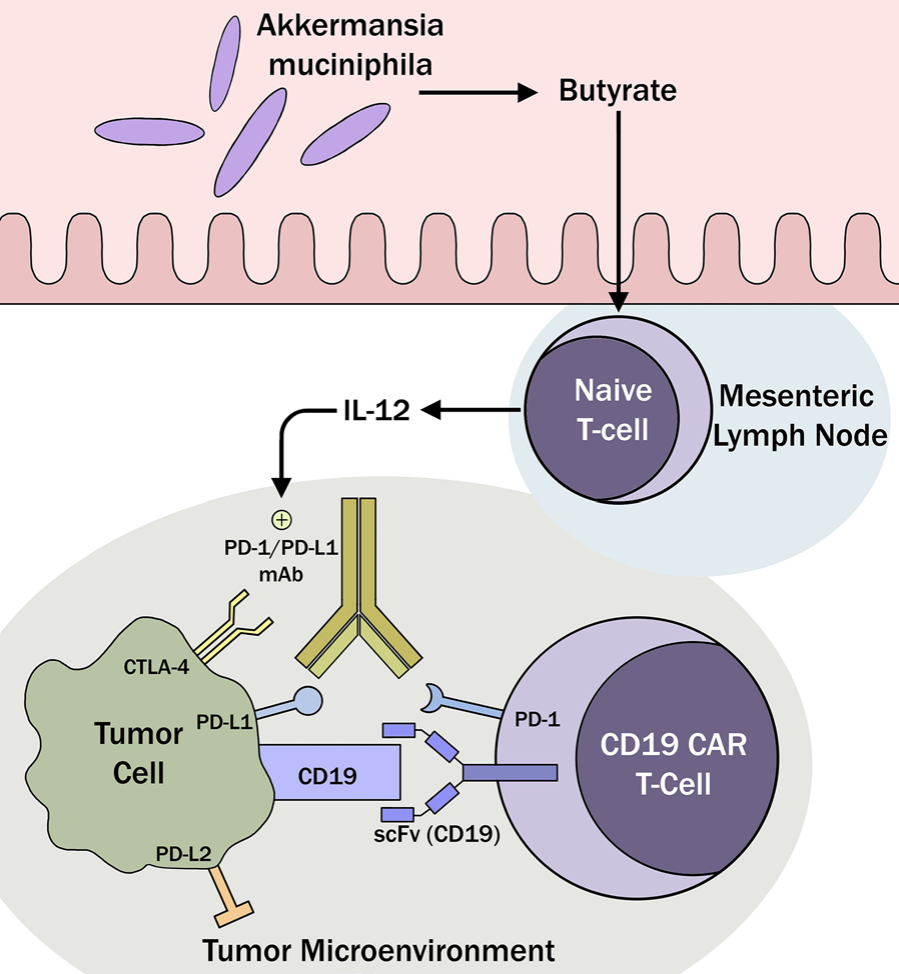
Butyrate produced by *Akkermansia muciniphila* would preferentially mount a pro-inflammatory immune response and suppress Tregs in the TME. This IL-12-mediated immune effector T-cell activation will boost ICI efficacy and secondarily enhance responses to CAR T-cells

Pre-clinical investigations and early-stage clinical data, that either used a combination of PD-1 blockade and CAR T-cells or those CAR T-cells that were engineered to secrete anti-PD-1-antibody (“built-in CAR T-cells”), have already demonstrated superior efficacy and increased persistence of CAR T-cells [39, 40]. For instance, there is an active clinical trial of CD19+ CAR T-cells with cell-intrinsic PD-1 inhibition by incorporation of a PD-1 shRNA-expressing cassette in the CAR lentivector in the setting of R/R B-cell lymphoma (NCT03208556). Through modulation of this immunosuppressive TME via enhancing ICI, strategic gut microbiota modulation may hold promise to bolster the potency of CAR T-cells as well by preventing CAR T-cell exhaustion and abrogating immunosuppressive TME, as discussed previously in “Pre-CAR conditioning and the role of antibiotics” section [23–25] and depicted in the hypothetical model (Fig. 2).

### A potential alternative to genome editing and programmable DNA cutters

With the incorporation of CRISPR/Cas9 and other genome-editing strategies [transcription activator-like effector nucleases (TALENs), etc.] into synthetic biology, increasingly sophisticated and specific CAR T-cells are being designed for clinical usage that will possess a knockout of a multiplex of inhibitory proteins [41–46]. Some of the inhibitory molecules exploited thus far include PD1, CTLA-4, TIM-3, LAG-3, Fas, and β-2 microglobulin, and intrinsic T-cell inhibitory enzymes including SHP-1 and DGK. Universal or ‘off-the-shelf’ CAR T-cells have further been created, for easy accessibility, by knocking out HLA and endogenous TCR.

For instance, Qasim et al. [43] successfully bridged 2 infants with R/R ALL to alloHCT by treating with a single dose of universal CD19+ CAR T-cells developed by lentiviral transduction and concurrent TALEN-mediated gene editing of TCR [deletion of T-cell receptor alpha constant (TRAC)]. With simultaneous editing of TCR, or CD52 to naturally prevent GVHD without the need for long-acting lympholytic antibodies, universal CD19+ CAR T-cells were used to treat R/R ALL. The clinical application of universal CAR T-cells has been reviewed by Zhao et al. [47]. Two phase-I multi-center trials are currently recruiting that utilize this method in pediatric (NCT02808442) and adult (NCT02746952) patients with ALL. Similarly, Eyquem et al. [42] designed a CAR T-cell with knocked out TRAC via CRISPR/Cas9. Zhang et al. [45] produced CD19+ CAR-T cells with LAG-3 knockout using CRISPR/Cas9-mediated gene editing. Ren et al. [41] also designed potent, universal CAR T-cells with knockout inhibitory ligands, including PD-1, utilizing CRISPR/cas9 multiplex gene-editing and showed an enhanced efficacy of CAR T-cells in tumor mice model. Similarly, Rupp et al. and Jung et al. [44, 46] generated CD19+ CAR T-cells via CRISPR/cas9 with PD1 and DGK knockouts respectively.

Several clinical trials are currently underway analyzing the efficacy of CRISPR/Cas9-mediated PD-1 knocked out CAR T-cells, both in HM and solid tumors (NCT03545815, NCT03298828). Although hypothetical currently, growing clinical evidence of gut microbiota manipulation to enhance responses to ICI may render a viable alternative to genome editing-based knockout of inhibitory molecules. This hypothesis is based on the shared immunological impact of gut microbiota and genome editing.

### CARs co-expressing cytokines, aka TRUCKs

T-cells redirected for universal cytokine-mediated killing (TRUCKs) are the fourth generation of CAR T-cells that are designed to engage allied immune effector cells by cytokine production within the TME. This is particularly efficacious in solid tumors as these employ complex immune evasion strategies in the tumor bed and localized delivery of cytotoxic cytokines via TRUCKs averts systemic toxicity.

Chmielewski et al. [48] first demonstrated effective in vitro tumor kill when CAR T-cells were engineered to release IL-12 (i.e. TRUCK) that recruited macrophages locally within the TME. This finding was further extrapolated to co-expression of other cytokines with TRUCKs. Carroll et al. [49] had earlier shown differential functions of human IL-18 on T-cell subsets in xenograft mice models: IL-18 enhanced the engraftment of cytotoxic T-cells (CD8+ T-cells) whereas it suppressed Tregs. Overall, this promoted the development of GVHD. Hu et al. [50] then harnessed this finding to design a universal CAR T-cell that co-expressed IL-18 and achieved enhanced tumor control in the melanoma mice model. Chmielewski et al. [51] then used this IL-18-inducing TRUCK construct to develop a CEA-directed CAR T-cell that resulted in improved antitumor activity in pancreatic mice model.

In a separate study, Tanoue et al. [52] recently characterized the critical role of IFNγ-expressing CD8+ T-cells in adenocarcinoma, gnotobiotic mice models treated with PD-1 inhibition and identified specific taxa, predominantly from the Firmicutes phylum, that acted in unison to suppress tumor growth.

Taken together, the gut microbiome may also hold potential to achieve similar results as those obtained by such sophisticated TRUCKs: specific gut taxa have also shown the capability to differentially activate effectors T-cells—Th17, CD8+ T-cells and Tregs—and induce cytokines, in early, pre-clinical studies.

### Novel strategies for enhancing CARs-orally delivered enzymes that protect the gut microbiota

Novel orally delivered beta-lactamases, intended to degrade β-lactam antibiotics in the upper GI tract to protect the gut microbiome, have shown the ability to limit the emergence of antimicrobial resistance (AMR) and reduce *Clostridioides difficile* infection (CDI) when given concurrently with systemic antibiotics. Specifically, in a Phase IIb clinical trial, SYN-004 (ribaxamase), a novel β-lactamase engineered to degrade higher-generation cephalosporins and penicillins, was demonstrated to be effective in curtailing AMR and CDI in patients treated with intravenous (IV) ceftriaxone for a lower respiratory infection [53, 54]. SYN-006, an oral carbapenemase, currently at the pre-clinical stage, protected the gut microbiome and reduced AMR in a porcine model of ertapenem-mediated dysbiosis [55]. Moreover, an oral formulation of the intestinal isozyme of alkaline phosphatase (IAP or SYN-020) has been shown in preclinical models to detoxify GI inflammatory mediators, improve gut barrier integrity, and promote the growth of commensal microbiota [56]. By protecting and preserving the normal microbiome, these enzymes could potentially have a substantial clinical impact on the overall spectrum of CAR T-cell therapy.

The beta-lactamases administered orally with IV broad-spectrum beta-lactams would be particularly beneficial in patients with hematological malignancies undergoing active treatment as they receive prophylactic antibiotics as well as broad-spectrum therapeutic antibiotics frequently. Both the beta-lactamases and IAP are anticipated to have favorable safety profiles and, with the ability to safeguard healthy gut taxa, have the potential to improve patient outcomes across the entire spectrum of cancer therapeutics, in general, and with CAR T-cells, specifically.

## Future directions in gut microbiome and CAR T-cells

There is growing pre-clinical evidence that delineates differential roles of the gut microbiota and allows their stratification with potentially stimulatory and inhibitory properties towards engineered CAR T-cells. Albeit the results from clinical studies in the ICI setting do not uniformly stratify “favorable” vs “unfavorable” gut microbial taxa, certain conclusions may yet be drawn (Table 1, adapted from Abid [10]). The bacteria belonging to the *Firmicutes* phylum [e.g. *Faecalibacterium* (*Faecalibacterium prausnitzii*), *Ruminococcacea*, *Clostridia*, etc.] are associated with good response and superior survival whereas the phylum *Proteobacteria* (e.g. *Enterobacteriaceae*) is associated with poor response and inferior survival. *Akkermansia muciniphila* belonging to the phylum *Verrucomicrobia* is associated with a boost in response and potentially superior survival. The impact of *Bacteroidetes* and *Bifidobacteria* are dependent upon the species involved and will need to be tailored during studies. For instance, *Bifidobacteria longum* has been shown to boost response in ICIs whereas *Bifidobacteria bifidum* was shown to be immunosuppressive. In order to make responses to CAR T-cells more homogeneous, predictable and durable, human studies will be needed. These studies may be performed in the following ways:

**Table 1:** Major gut microbial taxa and their predominant influence on systemic immunity and response to immunotherapy. Adapted from Abid [10]

Gut taxa	Immune effect/treatment response	Study type	Cancer type
Predominately positive influence			
Firmicutes			
*Faecalibacterium prauznitzii*	Boosts effector T-cells and dampens T-regs	Humans	Melanoma
*Faecalibacterium* spp.	Increases efficacy of anti-CTLA-4 immunotherapy	Humans	Metastatic melanoma
*Eubacterium limosum* *Clostridiales* spp. *Ruminococcaceae* spp. *Phascolarctobacterium* spp.	Boost CD8+ T-cells and enhance anti-PD-1 responses	Cell line	Colorectal Adenocarcinoma Cell line (MC38)
Fusobacteria			
*Fusobacterium ulcerans* *Fusobacterium varium*	Boost CD8+ T-cells and enhance anti-PD-1 responses	Cell line	MC38
Verrucomicrobia			
*Akkermansia muciniphila*	Increase in memory T-cells and decrease in T-regs in the TME	Humans	Epithelial tumors
	Increases mucus layer of the gut to prevent lipopolysaccharides absorption	Humans	Epithelial tumors
Mixed influence			
Bacteroidetes			
*Bacteroides fragilis*	Increases efficacy of anti-CTLA-4 immunotherapy	Human/animal/cell line	Epithelial tumors
	Promotion of T-regs through polysaccharide-A	Humans	Healthy humans
	Higher IL-12 levels in transplant recipients	Animal/cell line	Cervical cancer
*Bacteroides thetaiotaomicron*	Increases efficacy of anti-CTLA-4 and anti-PD-1 immunotherapy	Humans	Melanoma
*Bacteroides* spp.	Inferior response of anti-CTLA-4 immunotherapy	Humans	Metastatic melanoma
Actinobacteria			
*Bifidobacterium longum*	Increases CD8+ T-cells	Animal/cell line	Melanoma
		Humans	Melanoma
*Bifidobacterium bifidum*	Induces naïve T-cell differentiation into T-regs and increases IL-10	Humans/in vitro	Healthy humans
	Increases the integrity of epithelial barrier		
Predominately negative influence			
Proteobacteria			
*Enterobacteriaceae*	Inferior response and survival	Humans	Pediatric cancers

### Retrospective studies

Large, academic medical centers typically treat refractory cancer patients with CAR T-cells and the information is stored in robust databases. The centers may also have ongoing stool studies (e.g. for *Clostridioides difficile* infections) that include hematological patients who received a variety of cancer treatments at different treatment time-points. These stool samples, if available for CAR T-cell patients, may be ideal to study to delineate “responders” vs “non-responders,” in a retrospective setting. Taxonomic profiling using 16S ribosomal RNA (16S) gene sequencing and whole-genome shotgun sequencing (WGS) would further stratify the bacterial taxa involved in each of the two cohorts of CAR T-cell patients. The influence of behavior, lifestyle, dietary habits, obesity, co-morbidities, recent use of antibiotics or probiotics may further be analyzed, to study potential association with response and survival.

### Prospective studies

Stool samples are representative of the colonic flora and can be collected from patients undergoing CAR T-cells treatment at baseline and at subsequent treatment intervals. The aforementioned diagnostic modalities can be employed to study the bacterial flora at any stage of illness/treatment. Inclusion criteria could be adults undergoing CAR T-cells treatment for any reason who are not precluded to receive interventions that would confound gut microbiome and study results. Patients can be randomized to receive dietary interventions that could include non-western diet, or high-fiber, low-carb, ketogenic diet or non-absorbable oligosaccharides contained in potato starch, administration of prebiotics or single- or multi-strain probiotics, administration of narrow-spectrum antibiotic targeting specific unfavorable taxa, or single-arm FMT from healthy donors. The study outcomes may include changes in the gut (fecal) microbiome profile from baseline (via 16S profiling), the persistence of CAR T-cells, changes in immune cell populations and/or cytokines in the TME (via flow cytometry, qPCR), response to treatment as well as objective survival rates. Radiological imaging, tumor biopsies, and blood samples will need to be collected at matched treatment time-points and responders vs non-responders can be defined based on the established response assessment criteria for the underlying hematological illnesses.

It is noteworthy that WGS is superior to the 16S amplicon-based method for bacteriome sequencing as the former renders enhanced detection sensitivity, specificity and increased detection of diversity [57, 58]. Although there are qualitative differences in the data that are obtained from 16S and WGS that make these two methods distinctively valuable, all future microbiome studies should be based on standardized WGS techniques for reasons discussed above. Further, the microbiome study proposals and biorepository sample collection protocols ought to be detailed, specifically relating to standardization of sample collection, storage, processing and sequencing methodologies [59, 60].

## Evolving evidence

The microbiome studies have not expanded to CAR T-cell or other types of novel immune-engaging therapy yet and the earliest evidence of the role of antibiotics in ACT setting has been conflicting. The only one available thus far is a single-center observational study performed by van den Brink and colleagues. The study included 25 patients who received CAR T-cells with varying conditioning regimens and showed that responders had distinctive baseline microbiota composition as compared to non-responders. Interestingly, non-responders also did not experience toxicity and the microbiota in patients from both cohorts belonged to the Firmicutes phylum. The small study corroborates our hypothesis discussed above and hints towards a possible association between baseline gut taxa and response to CAR T-cells [61]. Additionally, certain gut taxa have been shown to suppress Tregs and potentiate the effect of ACTs, akin to the impact of preconditioning [30]. Given the narrow spectrum of CAR T-cell usage, further studies will require collaborative, multicenter efforts. Studies are now being conducted at the Memorial Sloan Kettering Cancer Center and the Medical College of Wisconsin to analyze any potential association of gut microbiota diversity/composition and response to CAR T-cells.

The findings discussed above illustrate that controlled manipulation of the gut microbiome could potentially be a rather inexpensive strategy to substantially enhance responses to posh and engineered therapeutic modalities such as immunotherapy and CAR T-cells in the future. Further large-scale, prospective studies will delineate its role in a myriad of engineered cancer therapeutics.

## Conclusion

Dysbiosis can increase the representation of deleterious microbiota that produces harmful metabolites and antigens, leading to maladaptive immune responses. Strategically averting gut dysbiosis, preventing the loss of diversity during treatment, and maintaining desirable taxa are needed to augment response to cancer treatment. Although there are suggestions that certain genera may hold a uniformly positive effect on responses, there are no definite or optimal consortia of bacterial taxa established yet that could be used in trials as a ‘probiotic’. Randomized studies are being planned to study differential responses and survival in melanoma patients receiving immunotherapy with FMT from long-term immunotherapy responders and an oral pill containing an exclusive assortment of microbes that mimic key taxa, explored thus far, in collaboration with the industry.

Meanwhile, we recommend against using commercially available probiotics specifically during cancer treatment [62]. These are typically composed of one or two strains of bacteria, whose impact on systemic immunity is not established, that could dilute the native gut flora and potentially make it less diverse. We further recommend against injudicious usage of broad-spectrum antibiotics around the time of immunotherapy and CAR T-cells.

### Practice points/highlights

- Immunotherapy is relatively safer than conventional treatment and prolongs survival in a variety of tumors in the relapsed/refractory disease settings.
- CD19-directed CAR T-cells are FDA-approved for patients with R/R ALL and DLBCL and benefit patients with otherwise poor outcomes.
- Despite impressive outcomes in select patients, there remains significant heterogeneity in clinical responses to both immunotherapy and CAR T-cells.
- The diversity and composition of the gut microbiome influences response to immunotherapy, according to recent evidence.
- The role of the gut microbiome in ACT or CAR T-cell setting has not been explored.
- We hypothesize that the gut microbiome modulation carries the potential for enhancing CAR T-cell responses and this theory is based on:
  - Pre-clinical and growing clinical evidence elucidating escape mechanisms in CAR T-cell non-responders.
  - Clinical evidence of enhancement of immunotherapy responses with gut microbiota manipulation.
  - Shared immunological features between CAR T-cells and other types of immunotherapy.
  - Early clinical evidence of the potential role of IL-6/STAT3 signature in superior CAR T-cell responses.
  - Early data on the suppressive role of Tregs on ACTs.
- Broad-spectrum antibiotics during immunotherapy and CAR T-cells may lead to dysbiosis and inferior responses and survival.
- Although we are aware of certain taxa that carry ‘favorable’ versus ‘unfavorable’ impact on immunotherapy responses, a definite, rationally designed conglomerate of live bacteria is yet to be established for use in prospective trials.

### Research agenda

- Retrospective studies analyzing the association between gut microbial diversity/composition and CAR T-cell responses.
- Retrospective studies analyzing any potential association between broad- versus narrow-spectrum antibiotics and CAR T-cell responses.
- Retrospective studies analyzing the potential association between behavior, lifestyle, dietary habits, obesity, co-morbidities, and probiotics and CAR T-cell responses.
- Delineate the relationship between the gut microbiome and cytokine and immune cell infiltration within the TME.
- Establish a clear relationship between the gut microbiome and tumor genome, if one exists.
- Metabolomic profiling to identify microbial metabolites as ‘biomarkers’ of response.
- Multicenter, prospective, randomized trials analyzing the impact of diet, non-absorbable oligosaccharides contained in potato starch, FMT from immunotherapy and CAR T-cell responders, or a rationally designed probiotic-containing consortia of live bacteria that mimic favorable taxa.

## Data Availability

Not applicable.

## Abbreviations

ICI: immune checkpoint inhibition
TME: tumor microenvironment
Tregs: regulatory T-cells
ACT: adoptive T-cell transfer
CAR T-cells: chimeric antigen receptor T-cells
CRS: cytokine release syndrome
BiTE: bi-specific T-cell engagers
TRUCK: T-cells redirected for universal cytokine-mediated killing
CRISPR: clustered regularly interspaced short palindromic repeats
TALEN: transcription activator-like effector nucleases
WGS: whole-genome shotgun sequencing

## References

[1] Mahoney KM, Rennert PD, Freeman GJ (2015). Combination cancer immunotherapy and new immunomodulatory targets. Nat Rev Drug Discov.

[2] Kuehn BM (2017). The promise and challenges of CAR-T gene therapy. JAMA.

[3] Hodi FS, O’Day SJ, McDermott DF (2010). Improved survival with ipilimumab in patients with metastatic melanoma. N Engl J Med.

[4] Topalian SL, Hodi FS, Brahmer JR (2012). Safety, activity, and immune correlates of anti-PD-1 antibody in cancer. N Engl J Med.

[5] Ansell SM, Lesokhin AM, Borrello I (2015). PD-1 blockade with nivolumab in relapsed or refractory Hodgkin’s lymphoma. N Engl J Med.

[6] June CH, Sadelain M (2018). Chimeric antigen receptor therapy. N Engl J Med.

[7] Schadendorf D, Hodi FS, Robert C (2015). Pooled analysis of long-term survival data from phase II and phase III trials of ipilimumab in unresectable or metastatic melanoma. J Clin Oncol.

[8] Robert C, Long GV, Brady B (2015). Nivolumab in previously untreated melanoma without BRAF mutation. N Engl J Med.

[9] Park JH, Riviere I, Gonen M (2018). Long-term follow-up of CD19 CAR therapy in acute lymphoblastic leukemia. N Engl J Med.

[10] Abid MB (2019). Could the menagerie of the gut microbiome really cure cancer? Hope or hype. J Immunother Cancer.

[11] Yi M, Yu S, Qin S (2018). Gut microbiome modulates efficacy of immune checkpoint inhibitors. J Hematol Oncol.

[12] Selby MJ, Engelhardt JJ, Quigley M (2013). Anti-CTLA-4 antibodies of IgG2a isotype enhance antitumor activity through reduction of intratumoral regulatory T cells. Cancer Immunol Res.

[13] Carbonnel F, Soularue E, Coutzac C (2017). Inflammatory bowel disease and cancer response due to anti-CTLA-4: is it in the flora?. Semin Immunopathol.

[14] Romano E, Kusio-Kobialka M, Foukas PG (2015). Ipilimumab-dependent cell-mediated cytotoxicity of regulatory T cells ex vivo by nonclassical monocytes in melanoma patients. Proc Natl Acad Sci USA.

[15] Tarhini AA, Zahoor H, Lin Y (2015). Baseline circulating IL-17 predicts toxicity while TGF-beta1 and IL-10 are prognostic of relapse in ipilimumab neoadjuvant therapy of melanoma. J Immunother Cancer.

[16] Callahan MK, Yang A, Tandon S (2011). Evaluation of serum IL-17 levels during ipilimumab therapy: correlation with colitis. J Clin Oncol.

[17] Li S, Zhang J, Wang M (2018). Treatment of acute lymphoblastic leukaemia with the second generation of CD19 CAR-T containing either CD28 or 4-1BB. Br J Haematol.

[18] Abid MB (2018). The revving up of CARs. Gene Ther.

[19] Maude SL, Laetsch TW, Buechner J (2018). Tisagenlecleucel in children and young adults with B-cell lymphoblastic leukemia. N Engl J Med.

[20] Neelapu SS, Locke FL, Bartlett NL (2017). Axicabtagene ciloleucel CAR T-cell therapy in refractory large B-cell lymphoma. N Engl J Med.

[21] Viaud S, Saccheri F, Mignot G (2013). The intestinal microbiota modulates the anticancer immune effects of cyclophosphamide. Science.

[22] Paulos CM, Wrzesinski C, Kaiser A (2007). Microbial translocation augments the function of adoptively transferred self/tumor-specific CD8+ T cells via TLR4 signaling. J Clin Invest.

[23] Xu X, Zhang X (2015). Effects of cyclophosphamide on immune system and gut microbiota in mice. Microbiol Res.

[24] Uribe-Herranz M, Bittinger K, Rafail S (2018). Gut microbiota modulates adoptive cell therapy via CD8α dendritic cells and IL-12. JCI Insight.

[25] Routy B, Gopalakrishnan V, Daillere R, Zitvogel L, Wargo JA, Kroemer G (2018). The gut microbiota influences anticancer immunosurveillance and general health. Nat Rev Clin Oncol.

[26] Routy B, Le Chatelier E, Derosa L (2018). Gut microbiome influences efficacy of PD-1-based immunotherapy against epithelial tumors. Science.

[27] Vetizou M, Pitt JM, Daillere R (2015). Anticancer immunotherapy by CTLA-4 blockade relies on the gut microbiota. Science.

[28] Kuczma MP, Ding ZC, Li T (2017). The impact of antibiotic usage on the efficacy of chemoimmunotherapy is contingent on the source of tumor-reactive T cells. Oncotarget.

[29] Akalin IPS, Angelis BD (2009). Effects of chimeric antigen receptor (CAR) expression on regulatory T cells. Mol Ther.

[30] Duell J, Dittrich M, Bedke T (2017). Frequency of regulatory T cells determines the outcome of the T-cell-engaging antibody blinatumomab in patients with B-precursor ALL. Leukemia.

[31] Fraietta JA, Lacey SF, Orlando EJ (2018). Determinants of response and resistance to CD19 chimeric antigen receptor (CAR) T cell therapy of chronic lymphocytic leukemia. Nat Med.

[32] Omenetti S, Pizarro TT (2015). The Treg/Th17 axis: a dynamic balance regulated by the gut microbiome. Front Immunol.

[33] Vignali DA, Collison LW, Workman CJ (2008). How regulatory T cells work. Nat Rev Immunol.

[34] Ruella M, Maus MV (2016). Catch me if you can: leukemia escape after CD19-directed T cell immunotherapies. Comput Struct Biotechnol J.

[35] Majzner RG, Mackall CL (2018). Tumor antigen escape from CAR T-cell therapy. Cancer Discov.

[36] Galon J, Rossi J, Turcan S (2017). Characterization of anti-CD19 chimeric antigen receptor (CAR) T cell-mediated tumor microenvironment immune gene profile in a multicenter trial (ZUMA-1) with axicabtagene ciloleucel (axi-cel, KTE-C19). J Clin Oncol.

[37] John LB, Kershaw MH, Darcy PK (2013). Blockade of PD-1 immunosuppression boosts CAR T-cell therapy. Oncoimmunology.

[38] Kasakovski D, Xu L, Li Y (2018). T cell senescence and CAR-T cell exhaustion in hematological malignancies. J Hematol Oncol.

[39] Cherkassky L, Morello A, Villena-Vargas J (2016). Human CAR T cells with cell-intrinsic PD-1 checkpoint blockade resist tumor-mediated inhibition. J Clin Invest.

[40] Maude SL, Hucks GE, Seif AE (2017). The effect of pembrolizumab in combination with CD19-targeted chimeric antigen receptor (CAR) T cells in relapsed acute lymphoblastic leukemia (ALL). J Clin Oncol.

[41] Ren J, Liu X, Fang C, Jiang S, June CH, Zhao Y (2017). Multiplex genome editing to generate universal CAR T cells resistant to PD1 inhibition. Clin Cancer Res.

[42] Eyquem J, Mansilla-Soto J, Giavridis T (2017). Targeting a CAR to the TRAC locus with CRISPR/Cas9 enhances tumour rejection. Nature.

[43] Qasim W, Zhan H, Samarasinghe S (2017). Molecular remission of infant B-ALL after infusion of universal TALEN gene-edited CAR T cells. Sci Transl Med.

[44] Rupp LJ, Schumann K, Roybal KT (2017). CRISPR/Cas9-mediated PD-1 disruption enhances anti-tumor efficacy of human chimeric antigen receptor T cells. Sci Rep.

[45] Zhang Y, Zhang X, Cheng C (2017). CRISPR-Cas9 mediated LAG-3 disruption in CAR-T cells. Front Med.

[46] Jung I-Y, Kim Y-Y, Yu H-S, Kim S, Thon JN, Lee J (2017). CRISPR/Cas9-mediated diacylglycerol kinase knockout potentiates anti-tumor efficacy of human chimeric antigen receptor T-cells. Blood.

[47] Zhao J, Lin Q, Song Y, Liu D (2018). Universal CARs, universal T cells, and universal CAR T cells. J Hematol Oncol.

[48] Chmielewski M, Kopecky C, Hombach AA, Abken H (2011). IL-12 release by engineered T cells expressing chimeric antigen receptors can effectively Muster an antigen-independent macrophage response on tumor cells that have shut down tumor antigen expression. Can Res.

[49] Carroll RG, Carpenito C, Shan X (2008). Distinct effects of IL-18 on the engraftment and function of human effector CD8 T cells and regulatory T cells. PLoS ONE.

[50] Hu B, Ren J, Luo Y (2017). Augmentation of antitumor immunity by human and mouse CAR T cells secreting IL-18. Cell Rep.

[51] Chmielewski M, Abken H (2017). CAR T cells releasing IL-18 convert to T-Bet(high) FoxO1(low) effectors that exhibit augmented activity against advanced solid tumors. Cell Rep..

[52] Tanoue T, Morita S, Plichta DR (2019). A defined commensal consortium elicits CD8 T cells and anti-cancer immunity. Nature.

[53] Kokai-Kun JF, Roberts T, Coughlin O (2017). The oral beta-lactamase SYN-004 (ribaxamase) degrades ceftriaxone excreted into the intestine in phase 2a clinical studies. Antimicrob Agents Chemother.

[54] SYN-004 (ribaxamase) protects the gut microbiome of patients treated with ceftriaxone from disruption and reduces the emergence of antimicrobial resistance. Presented at ID week 2018; October 4, 2018; San Francisco, California. Abstract 1337.

[55] SYN-006, a novel carbapenemase, intended to protect the gut microbiome from antibiotic-mediated damage may also reduce propagation of carbapenem-resistant pathogens. Presented at ID week 2018; October 4, 2018; San Francisco, California. Abstract 630.

[56] Alam SN, Yammine H, Moaven O (2014). Intestinal alkaline phosphatase prevents antibiotic-induced susceptibility to enteric pathogens. Ann Surg.

[57] Laurence M, Hatzis C, Brash DE (2014). Common contaminants in next-generation sequencing that hinder discovery of low-abundance microbes. PLoS ONE.

[58] Ballester LY, Luthra R, Kanagal-Shamanna R, Singh RR (2016). Advances in clinical next-generation sequencing: target enrichment and sequencing technologies. Expert Rev Mol Diagn.

[59] Kim D, Hofstaedter CE, Zhao C (2017). Optimizing methods and dodging pitfalls in microbiome research. Microbiome.

[60] Goodrich JK, Di Rienzi SC, Poole AC (2014). Conducting a microbiome study. Cell.

[61] Smith M, Littmann E, Slingerland J (2019). Intestinal microbiome analyses identify biomarkers for patient response to CAR T cell therapy. Biol Blood Marrow Transplant.

[62] Abid MB, Koh CJ (2019). Probiotics in health and disease: fooling mother nature?. Infection.

